# Sometimes Less Is More: Perinatal Bacterial Exposure May Be More Important than Hepatitis for Liver Tumor Development

**Published:** 2008-10

**Authors:** David A. Taylor

*Helicobacter hepaticus*, a bacterium discovered in 1994 and widespread in many experimental mouse populations, is associated with a high incidence of liver tumors in aging mice. A new mouse study shows that perinatal exposure to this pathogen, rather than development of hepatitis itself, may be the single most important factor in the development of liver tumors caused by *H. hepaticus* **[*EHP* 116:1352–1356; Diwan et al.]**. The results support evidence from other studies that progressive hepatitis and liver tumors in older mice may stem from early-life exposure.

The researchers isolated a strain of *H. hepaticus* from infected A/J mice. Female mice received injections with a single dose of the bacteria. Females testing positive for the bacteria were bred with uninfected males. The researchers assessed liver histopathologic findings and tumor growth in male offspring aged 2 weeks to 2 years. Uninfected weanling males from another lab were injected at 3–4 weeks.

The results showed a significant incidence of liver tumors in the offspring after intraperitoneal maternal exposure to the bacteria: 33% developed liver tumors, usually multiple tumors, and 18% developed hepatocellular carcinoma. None of the mice injected with the bacteria as young adults developed any tumors.

Another striking result was that tumor outcome was not closely linked to severity of hepatitis; mice that contracted hepatitis did not necessarily develop hepatic tumors. Rather, it appeared that early exposure to the bacteria, not the hepatitis itself, was key to fostering tumor growth.

The type of additional perinatal event required to induce tumor development is unknown but could involve DNA damage at that vulnerable early stage, followed by subacute inflammation of the liver in response to *H. hepaticus* infection. The authors note that similar scenarios could apply to human infection with *Helicobacter pylori* and other pathogens linked with cancer. The results point to the need for further study of changes in perinatal tissues in response to such infections.

